# Differential outcome of neurological HCMV infection in two hematopoietic stem cell transplant recipients

**DOI:** 10.1186/1471-2334-12-238

**Published:** 2012-10-03

**Authors:** Anna Amelia Colombo, Giovanna Giorgiani, Vanina Rognoni, Paola Villani, Milena Furione, Mario Regazzi Bonora, Emilio Paolo Alessandrino, Marco Zecca, Fausto Baldanti

**Affiliations:** 1 Centro Trapianti di Midollo Osseo, Istituto di Ematologia, Fondazione IRCCS Policlinico San Matteo, Pavia, Italy; 2 Struttura Complessa di Ematologia ed Oncologia Pediatrica, Fondazione IRCCS Policlinico San Matteo, Pavia, Italy; 3 Struttura Semplice Virologia Molecolare, Struttura Complessa Virologia e Microbiologia, Fondazione IRCCS Policlinico San Matteo, Pavia, Italy; 4 Dipartimento di Farmacologia, Fondazione IRCCS Policlinico San Matteo, Pavia, Italy

**Keywords:** HCMV, CNS, T-cell depleted, HSCT

## Abstract

**Background:**

Human cytomegalovirus (HCMV) infection of the central nervous system (CNS) is a rare but life threatening condition which may follow hematopoietic stem cell transplantation. Diagnosis, monitoring and treatment approaches rely on anecdotal reports.

**Case presentations:**

The different outcomes of HCMV CNS disease in an adult and a pediatric T-cell depleted hematopoietic stem cell transplant (HSCT) recipient are reported. In the first case, HCMV encephalitis emerged in the context of simultaneous impairment of the T- and B-cell immunity. Antiviral treatment only reduced viral load in peripheral blood and the patient died. In the second case, an HCMV radiculopathy was observed and antiviral treatment was adjusted on the basis of intrathecal drug level. In addition, donor HCMV-specific cytotoxic T lymphocytes (CTLs) were infused. Viral load in the CNS decreased and the patient recovered from the acute event. In neither case were drug-resistant HCMV variants observed in blood or CNS samples.

**Conclusions:**

T-cell depleted HSCT appears a predisposing condition for CNS HCMV infection since never observed in other HSCT recipients at our center in the last 15 years. Intensive diagnostic approaches and timely aggressive combination treatments might improve clinical outcome in these patients.

## Background

Over the last 15 years at our center roughly 800 pediatric and 400 adult patients have received a hematopoietic stem cell transplant (HSCT). Recently, T-cell depletion has been introduced both as a graft manipulation before infusion from haploidentical donors and as an *in vivo* T-cell depletion in matched unrelated HSCT. While these procedures are not routinely performed, the number of T-cell depleted HSCT has steadily increased over the last 5 years and consisting now about 25% of all pediatric and 60% of all adult HSCT performed at out Instition.

It should be noted that, depletion of T-cells before graft infusion or in the post transplant period reduces the risk for graft-versus-host disease (GVHD), but also increases the risk for infectious complications [1,2].

Human Cytomegalovirus (HCMV) infections of the central nervous system (CNS) are rare but life-threatening complications following HSCT [3,4]. The high mortality rate has been associated with immune system impairment and reduced efficacy of antiviral treatment due to the poor bioavailability of ganciclovir (GCV) and foscarnet (PFA) in cerebrospinal fluid (CSF) [5,6]. We report on the different outcomes of CNS HCMV infection in two T-cell depleted HSCT recipients.

## Case presentations

### Virologic monitoring and treatment

All T-cell depleted HSCT recipients undergo frequent virologic monitoring associated with pre-emptive treatment protocols for most common viral infections, such as HCMV [7], EBV [8,9], and adenovirus [10].

In more detail, in the absence of active HCMV infection or GVHD, real-time PCR for HCMV DNA quantification in whole blood [11,12] is performed once a week in the first three months post-transplant, once every two weeks in the next three months and once every four weeks in the next six months. In the presence of active HCMV infection or GVHD, real-time PCR is performed twice a week. Treatment with GCV was initiated upon detection of 10,000 HCMV DNA copies in whole blood [12,13].

The emergence of GCV- and PFA-resistant HCMV strains is monitored by sequencing HCMV UL97 and UL54 genes [14].

In all pediatric patients receiving haploidentical HSCT, HCMV- EBV- and adenovirus- specific donor derived CTLs are generated before transplantation and administered in case of refractory infections [10,15].

### Patient no. 1

**Figure 1:**
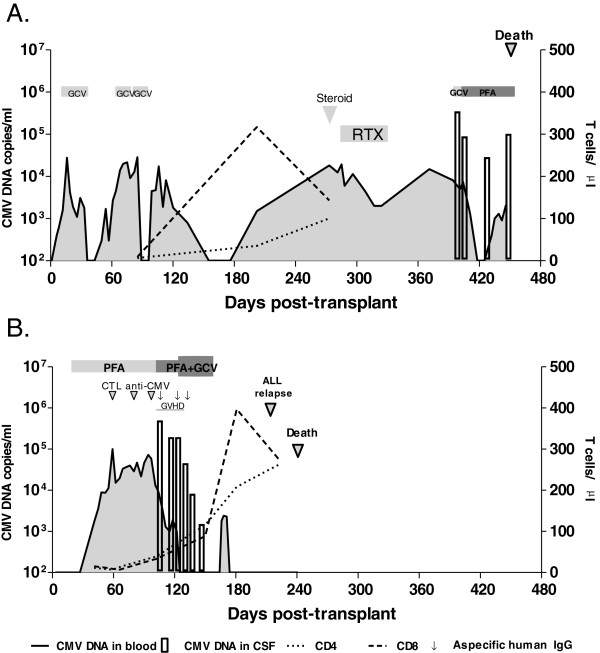
**Virologic monitoring of CNS HCMV infection in (A) an adult HSCT and (B) in a pediatric HSCT recipient.** The sensitivity of the in-house developed HCMV DNA Real-Time PCR is 100 copies/mL for blood specimens and 20 copies/mL for CSF specimens.

A HCMV-seropositive 58 year old man with high-risk acute myeloid leukemia received a matched unrelated HSCT from a HCMV-seronegative donor. Transplant conditioning included 200 cGy total body irradiation (TBI), fludarabine, alemtuzumab and melphalan. Cyclosporine and a short course of methotrexate were given as prophylaxis against GVHD. After engraftment, the patient presented with three recurrent asymptomatic HCMV DNAemia episodes (>10,000 copies/mL) and pre-emptive treatment (GCV 5 mg/kg/twice a day) was administered at days 15–35, days 69–74 and days 85–93. Nine months after transplant, prednisone (50 mg/Kg/once a day), polyclonal immunoglobulins (400 mg/Kg/ every four days) and rituximab (RTX) (600 mg/once a week) were administered to treat thrombocytopenia (PLT 14.000/μL) in the presence of antibodies to platelet membrane glycoproteins (GPIb/IX and GP IIb/IIIa). On day 396, two months after the last of four RTX doses, the patient showed progressive memory deficit, temporal disorientation, astenia and weight loss. Expansion of the NK cell subset (1,449 cells/μL), reduced CD4 (132 cells/μL) and CD8 (79.5 cells/μL) T-cell counts and depletion of CD19 cells (0 cells/μL) were observed. Brain Magnetic Resonance Imaging (MRI) showed several foci of restricted diffusion along the ventricles and the ependyma, consistent with encephalitis. Despite blood brain barrier damage (albumin 877 mg/L CSF), a higher HCMV DNA level (346,780 copies/mL) in CSF than in blood (8,100 copies/mL) was observed. GCV treatment (5 mg/kg/ twice a day) was initiated. On day 407, the emergence of a GCV-resistant HCMV strain was hypothesized based on fever and dyspnoea: thus, GCV was empirically substituted with PFA (90 mg/kg/ twice a day). On day 418, HCMV DNA became undetectable in blood, but persisted at a high level in CSF (88,920 copies/mL) and PFA treatment was supplemented with anti-HCMV immunoglobulins. Meanwhile, *Staphylococcus epidermidis* was isolated in a blood culture. Subsequently, teicoplanin therapy was associated with disappearance of fever and dyspnoea. Sequencing of HCMV UL97 and UL54 showed the absence of drug-resistant HCMV strains in blood or CSF. However, the patient experienced progressive deterioration in neurological function, accompanied by a worsening of the brain MRI. On day 449 after transplantation, HCMV DNA persisted at a high level in CSF (102,860 copies/mL) in parallel with severe blood brain barrier damage (albumin 1,310 mg/L). Severe lymphopenia with a predominance of NK cells (228 cells/μL), low numbers of CD8 (5.4 cells/μL) and CD4 T-cells (8.5 cells/μL), and a complete absence of B-cells was observed. Two days later, the patient became unresponsive, developed fever and eventually died. The virologic follow-up is summarized in Figure 1A.

### Patient no. 2

A HCMV-seronegative 4 year old girl who received a haploidentical T-cell depleted HSCT from a HCMV-seropositive donor to treat an acute lymphoblastic leukaemia (ALL), which had relapsed five months before. Transplant conditioning included TBI, fludarabine, thiotepa and antithymocyte globulin. No GVHD prophylaxis was administered. After engraftment, on day 24, an HCMV infection was detected in blood (2 pp65-positive cells/200,000 cells) and treatement with PFA (90 mg/kg/twice a day) was initiated. Despite antiviral treatment, HCMV viral load increased (100,500 DNA copies/mL blood on day 59) and donor HCMV-specific CTLs were infused on days 59, 80 and 97. From day 97, GCV (5 mg/kg/ twice a day) was added to PFA. Persistent fever was present from day 90. On day 102, very low CD4 (39 cells/μL) and CD8 (33 cells/μL) T-cell counts were observed. On day 105, cutaneous GVHD was diagnosed, and methylprednisolone (1.5 mg/kg/once a day) was administered, followed by tacrolimus (1 g/once a day) and three doses of polyclonal immunoglobulin. On day 106, the patient reported acute cervical and right knee pain, accompanied by fever, asthenia and headache. X-ray and CT scan did not show skeletal or neurologic trauma. Tramadol hydrochloride (15 mg intravenously), ketorolac tromethamine (8 mg intravenously) were ineffective in controlling pain and morphine (2 mg intravenously) was infused on day 106. A high HCMV DNA load (495,900 copies/mL) was detected in CSF in contrast with 9,200 HCMV DNA copies/mL in blood. Sequencing of HCMV UL97 and UL54 showed the absence of drug-resistant HCMV strains in blood or CSF. On day 114 the neurological picture improved, but two weeks later viral load was still elevated. GCV was undetectable in plasma, while it was present at a low basal concentration (0.55 μg/mL) in CSF. On day 132, GCV was administered at an increased dosage of 7.5 mg/kg twice a day. HCMV DNA load declined progressively until disappearance, initially in blood (day 132) and then in CSF (day 158). From day 150, T-lymphocyte reconstitution was observed (CD4, 115 cells/μL and CD8, 88 cells/μL, followed by CD4, 208 cells/μL and CD8, 397 cells/μL on day 181). Unfortunately, on day 214, the patient relapsed ALL and died one month later. The virologic follow-up is summarized in Figure 1B.

## Conclusion

Following allogeneic HSCT, HCMV end-organ localization is more commonly characterized by interstitial pneumonia and gastrointestinal disease, while HCMV encephalitis and radiculoneuropathy have been seldomly reported [3]. The pathogenesis of HCMV CNS disease is still debated. Recurrent viremic events may lead to end-organ neurologic syndromes. Sub-optimal CNS antiviral drug penetration along with impaired local immunosurveillance both represent causes of virus latency, uncontrolled viral replication and HCMV disease [6,16,17]. In our patients, this serious complication appeared in the context of persistent and severe T-cell depletion. In addition, in the adult patient, the immune impairment was worsened by CD20 B-cell depletion.

As also observed in other cases [4], HCMV CNS disease followed the failure of pre-emptive monotherapy to control disseminated HCMV. The emergence of a drug-resistant HCMV strain was suspected, but excluded in both cases. Rescue treatment was more aggressive in the pediatric patient, with the combined administration of PFA, high dose GCV and infusions of HCMV-specific CTL. The GCV dosage was increased due to negligible GCV levels in both blood and CSF. Timely HCMV-specific CTL infusions were possible since at our Institution viral-specific CTLs are generated in advance from the donor in all cases of haploidentical T-cell depleted pediatric transplantation. Unfortunately, for the adult patient the graft was from a bank donor and precluded the possibility to generate in advance specific CTLs.

These two cases confirm the severity of CNS HCMV disease in HSCT recipients. However, they also show that frequent monitoring and timely aggressive treatment interventions based upon combined administration of GCV and PFA and, whenever possible, HCMV-specific CTLs may alter the course of the disease. Plasmatic and intrathecal antiviral drug levels should be monitored in order to avoid treatment failure in the absence of drug-resistant HCMV strains.

In consideration of the increasing number of patients who will receive an *in vivo* or *in vitro* T-cell depleted transplant, new strategies should be urgently adopted to prevent prolonged HCMV immunodeficiency and the emergence of severe HCMV-related complications. New antiviral drugs [18], vaccines [19,20], and reconstitution of HCMV cellular immunity [21-23] should be considered when designing new models of HCMV disease prevention.

## Consent

Written informed consent was obtained from the two patients (from the parents in the pediatric case) for publication. A copy of the written consent is available for review by the Series Editor of this journal.

## Abbreviations

CNS: Central nervous system; CTL: Cytotoxic T lymphocytes; GCV: Ganciclovir; GVHD: Graft-versus-host disease; HCMV: Human cytomegalovirus; HSCT: Hematopoietic stem cell transplant; MRI: Magnetic resonance imaging; PFA: Foscarnet; TBI: Total body irradiation.

## Competing interests

The authors declare that they have no competing interests.

## Authors’ contributions

AAC, GG, PEA and MZ clinical case management and discussion. PV and MRB pharmacokinetics assessment. MF and VR virological analysis and case discussion. AAC, MZ and FB case discussion, data analysis and manuscript writing. FB fund raising. All authors discussed the data, read and approved the final manuscript.

## Pre-publication history

The pre-publication history for this paper can be accessed here:

http://www.biomedcentral.com/1471-2334/12/238/prepub
